# Effectiveness and safety of vedolizumab for ulcerative colitis: a single-center retrospective real-world study in China

**DOI:** 10.3389/fphar.2023.1188751

**Published:** 2023-05-04

**Authors:** Kaituo Huang, Jing Liu, Wenhao Xia, Chuwen Tian, Lingya Yao, Qian Cao, Haotian Chen

**Affiliations:** ^1^ Department of Gastroenterology, Sir Run Run Shaw Hospital, College of Medicine Zhejiang University, Hangzhou, China; ^2^ Inflammatory Bowel Disease Center of Sir Run Run Shaw Hospital, College of Medicine Zhejiang University, Hangzhou, China

**Keywords:** ulcerative colitis, vedolizumab, effectiveness, drug safety, real-world study, China

## Abstract

**Introduction:** The effectiveness and safety of vedolizumab (VDZ) against ulcerative colitis (UC) have been validated in several randomized controlled trials and real-world studies in Western countries. However, there are few studies on VDZ in Asia, and the follow-up period for these studies is generally short. Therefore, this study evaluates the long-term effectiveness and safety of VDZ in Chinese patients with UC.

**Methods:** This retrospective study included patients with moderate to severe UC treated with VDZ between September 2019 and April 2022 at Sir Run Run Shaw Hospital, College of Medicine Zhejiang University. Clinical response and remission were assessed using the patient reported outcomes and the partial Mayo Score, and mucosal remission and healing were assessed using the Mayo Endoscopy Score. The primary endpoint was defined as clinical remission at week 14, and secondary endpoints included clinical response and steroid-free clinical remission at week 14, clinical response, clinical remission, and steroid-free clinical remission at week 52, and mucosal remission and healing at weeks 14 ± 8 and 52 ± 8.

**Results:** Overall, 64 patients with moderate to severe UC were enrolled. The clinical response, clinical remission, and steroid-free clinical remission rates at week 14 were 73.4% (47/64), 65.6% (42/64), and 54.7% (35/64), respectively. Mucosal remission and healing rates at week 14 ± 8 were 64.7% (22/34) and 38.2% (13/34), respectively. A total of 48 patients were treated with VDZ for 52 weeks. Based on intention-to-treat analysis, the clinical response, clinical remission, and steroid-free clinical remission rates at week 52 were 68.8% (44/64), 64.1% (41/64), and 64.1% (41/64), respectively. Mucosal remission and healing rates at week 52 ± 8 were 70.6% (12/17) and 35.3% (6/17), respectively. During the follow-up period, the most common adverse event was skin rash (6/64). No cases of acute infusion reactions, delayed allergic reactions, new hepatitis B infections, active tuberculosis, or malignant tumors were reported.

**Conclusion:** In this single-center retrospective real-world study, the effectiveness of long-term use of VDZ for Chinese patients with UC was similar to the outcomes previously reported in other geographical regions and populations; no new safety signals were found compared with other registered studies.

## 1 Introduction

Ulcerative colitis (UC), a chronic inflammatory bowel disease (IBD), primarily affects the rectum and colon and is caused by environmental factors, genetic predisposition, immune dysregulation, and gut dysbiosis (Lee et al., 2018). UC often presents with persistent or recurrent episodes of diarrhea, mucopurulent stools, abdominal pain, rectal tenesmus, and varying degrees of systemic symptoms. The annual incidence of UC is increasing in Asia, especially in newly industrialized countries, placing a severe disease burden on patients and society (Park and Cheon, 2021).

Conventional therapeutic agents for UC include 5-aminosalicylates, immunomodulators, and corticosteroids. Biologics can be considered for patients with an inadequate response or intolerance to conventional therapies (Raine et al., 2022). Initially, only anti-tumor necrosis factor (TNF) agents (including infliximab [IFX], adalimumab [ADA], and golimumab) were approved for UC. However, up to a third of patients do not respond to anti-TNF agents (Argollo et al., 2020). Therefore, new biologics are urgently required for conversion therapy in these patients.

Vedolizumab (VDZ) is a humanized gut-specific monoclonal antibody that selectively binds to α4β7 integrin and inhibits its binding to mucosal addressin cell adhesion molecule-1, thereby reducing the migration of lymphocytes through the endothelium of the gut and reducing inflammation in intestinal tissues (Wyant et al., 2016). In the GEMINI randomized controlled trial (RCT), VDZ was superior to the placebo in inducing and maintaining clinical remission and mucosal healing and showed a favorable safety profile during follow-up (Feagan et al., 2013). Therefore, the US Food and Drug Administration and the European Medicines Agency approved VDZ for UC in May 2014. IFX and VDZ are the only biological agents that have been approved by the State Drug Administration of China for the treatment of UC; the latter was approved in March 2020. However, owing to explicit inclusion and exclusion criteria, patients participating in clinical trials may not adequately represent the entire patient population. Therefore, real-world studies (RWSs) have become as important as RCTs (Kim et al., 2018).

Although several RWSs have been conducted for VDZ (Kotze et al., 2018; Narula et al., 2018; White et al., 2020), they have primarily focused on Western populations. Meanwhile, a limited number of studies have been reported on the real-world effectiveness of VDZ among Asian patients with IBD; those that have been performed have only presented short-term results. Therefore, the current RWS was performed to evaluate the long-term effectiveness and safety of VDZ in Chinese patients with UC.

## 2 Materials and methods

### 2.1 Study design and population

This was a retrospective study conducted at the Inflammatory Bowel Disease Center of Sir Run Run Shaw Hospital, College of Medicine Zhejiang University. Patients with active UC at our center who were treated with VDZ between September 2019 and April 2022 were enrolled. The inclusion criteria were as follows: 1) ≥ 18 years of age, 2) diagnosed as having UC for ≥3 months, 3) moderate to severe disease activity (Mayo Score ≥6), and 4) had received at least three doses of intravenous VDZ. Ultimately, 64 patients were enrolled in this study.

VDZ was administered intravenously at a dose of 300 mg on weeks 0, 2, and 6 for the induction treatment and every 8 weeks thereafter for maintenance. Complete blood count, liver function tests, C-reactive protein (CRP) level, erythrocyte sedimentation rate, and other relevant laboratory tests were conducted before each infusion.

A combination of corticosteroids was permitted in the study. That is, prednisone was initially administered at a dose of 40 mg/day for 4 weeks. The dose was then reduced by 5 mg/day over 1 week intervals until reaching a dose of 20 mg/day (i.e., week 5: 35 mg/day, week 6: 30 mg/day, week 7: 25 mg/day, week 8: 20 mg/day), which was maintained for 4 weeks and then further reduced by 2.5 mg/day over 1 week intervals until prednisone was discontinued. The total treatment duration was 4–6 months.

### 2.2 Variables

Electronic medical records of the included patients were reviewed. Information including sex, age, body mass index, smoking history, duration of UC, extent of UC, disease activity, time of initiation and withdrawal from VDZ treatment, laboratory tests, endoscopic examinations, prior and concomitant drug use, extraintestinal manifestations, and adverse events was obtained.

### 2.3 Outcomes and definitions

The extent of UC was defined according to disease site: disease involving the rectum only (E1), disease distal to the splenic flexure (E2), and disease extending proximal to the splenic flexure (E3). Disease activity was classified as mild (3–5 points), moderate (6–10 points), or severe (11–12 points), according to the Mayo Score. During follow-up, patient-reported outcomes (PRO2, rectal bleeding, and stool frequency) and the partial Mayo Score (rectal bleeding, stool frequency, and physician’s global assessment) were used to assess the degree of disease control; higher scores indicated more active disease. The Mayo Endoscopic Score (MES) was used to assess the degree of mucosal lesions and was scored 0–3 according to severity.

The primary endpoint of this study was clinical remission at week 14. The secondary endpoints included clinical response and steroid-free clinical remission at week 14 and week 52, respectively, and mucosal remission and mucosal healing at weeks 14 ± 8 and 52 ± 8, respectively, during the follow-up. Clinical response was defined as a decrease of at least 50% in PRO2, and clinical remission as PRO2 (rectal bleeding = 0, stool frequency = 0) or a partial Mayo Score <3 with no subscore >1. Mucosal remission was defined as an MES ≤1, and mucosal healing as an MES = 0.

All patients were followed up from the time of VDZ infusion until either one of the following occurred: death, surgery, treatment discontinuation, or termination of the follow-up period. All scoring procedures were performed by the same physician to reduce unnecessary bias. All adverse events related to VDZ, including acute infusion reactions, delayed allergic reactions, acute and chronic infections, and malignancies, were recorded during treatment.

### 2.4 Statistical analysis

SPSS 26.0 was used for statistical analysis, and *p* < 0.05 was considered to indicate statistical significance. Normally distributed continuous variables were expressed as mean ± standard deviation, and *t-*tests were used for comparison between groups. Non-normally distributed continuous variables were expressed as median and interquartile range (IQR), and the Mann–Whitney *U* test was used for comparison between groups. Categorical variables were expressed as numbers and percentages (%), and the chi-square or Fisher’s exact test was used for comparison between groups. Per protocol analysis and intention-to-treat analysis were used to analyze the clinical response and remission rates at Week 52.

### 2.5 Ethical considerations

This study was approved by the ethical review committee of the Sir Run Run Shaw Hospital, College of Medicine Zhejiang University. All patients provided written informed consent.

## 3 Results

### 3.1 Baseline characteristics

**FIGURE 1:**
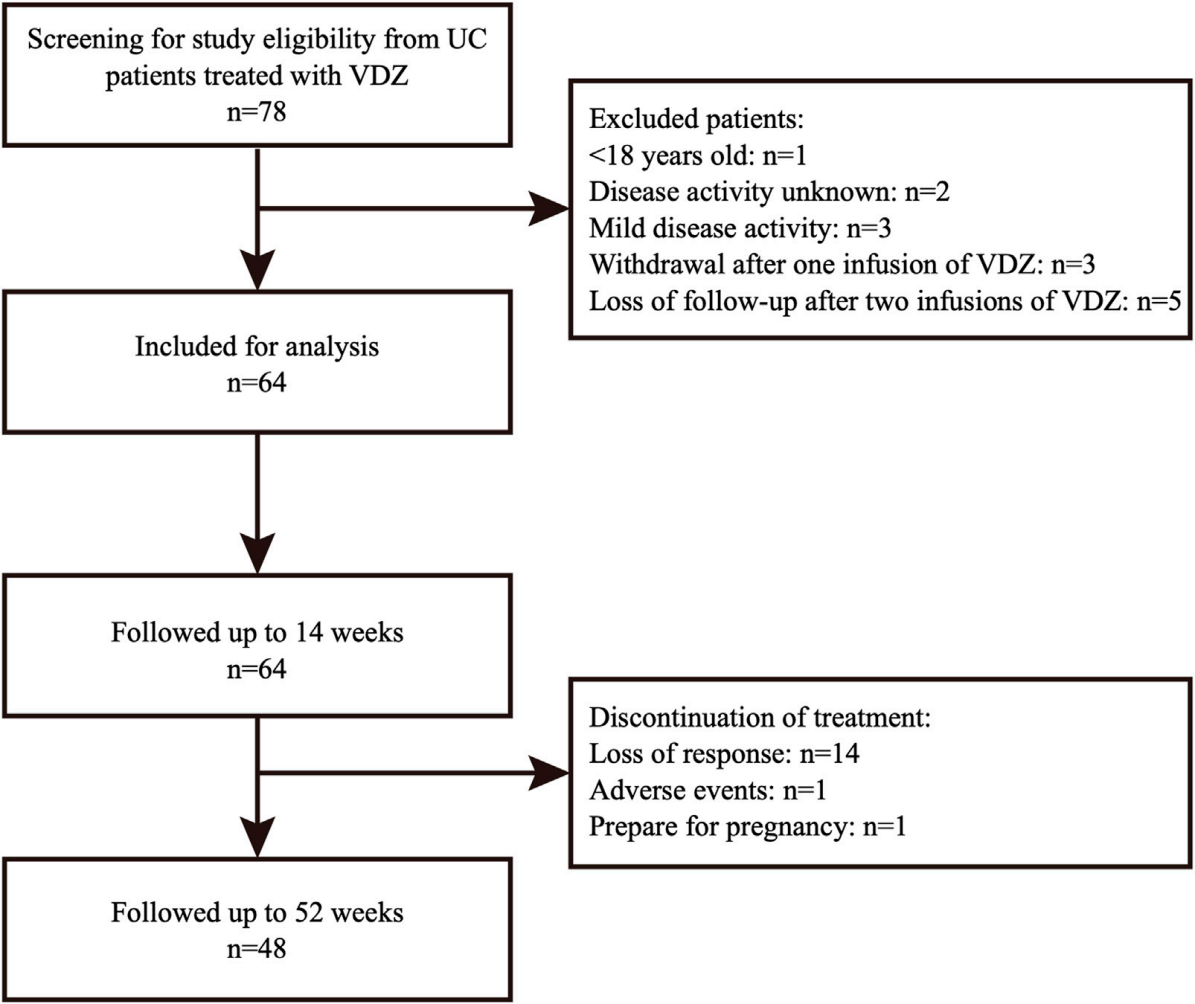
Study flowchart of patients included in analysis. UC, ulcerative colitis; VDZ, vedolizumab.

Between September 2019 and April 2022, 64 patients with moderate to severe UC met the inclusion criteria (Figure 1). The mean age of the patients was 45.1 ± 13.3 years, and 56.3% were males. The median disease duration was 5.3 (2.0, 8.9) years, and the median Mayo Score at baseline was 9.0 (8.0–10.0). Most patients had extensive (48.4%) and moderate (79.7%) disease activity. Before receiving VDZ, 33 (51.6%) patients had previously received corticosteroids, and 8 were still receiving corticosteroids at baseline. Additionally, 8 (12.5%) patients had received anti-TNF agents, all of which were eventually discontinued due to loss of response. An additional 4 (6.3%) patients had received tofacitinib (TOF); 2 were still receiving TOF at the start of VDZ treatment. At baseline, the mean albumin level was 38.8 (35.6, 41.8) g/L, while the median leukocyte count, hemoglobin and CRP levels, and erythrocyte sedimentation rate were within the normal range. Patient characteristics are shown in Table 1.

**TABLE 1:** Baseline clinical characteristics of patients with ulcerative colitis.

Clinical parameters		N = 64
Age, year		45.1 ± 13.3
Disease duration^a^, year		5.3 (2.0, 8.9)
BMI^b^, kg/m2		21.7 ± 3.1
Mayo Score		9.0 (8.0, 10.0)
Partial Mayo Score		7.0 (6.0, 8.0)
Sex, n (%)	Male	36 (56.3%)
	Female	28 (43.8%)
Smoking status, n (%)	Current smoker	3 (4.7%)
	Former smoker	4 (6.3%)
	Never smoked	57 (89.1%)
Disease extent, n (%)	E1	2 (3.1%)
	E2	29 (45.3%)
	E3	31 (48.4%)
	NA	2 (3.1%)
Disease activity, n (%)	Moderate	51 (79.7%)
	Severe	13 (20.3%)
Extraintestinal manifestations, n (%)	Skin manifestations	6 (9.4%)
	Joint manifestations	4 (6.3%)
Infections, n (%)	Latent tuberculosis infections	7 (10.9%)
	Hepatitis B infections	4 (6.3%)
Prior medications, n (%)	Corticosteroids	33 (51.6%)
	Anti-TNF agents	8 (12.5%)
	TOF	4 (6.3%)
Concomitant medications, n (%)	Corticosteroids	8 (12.5%)
	TOF	2 (3.1%)
Laboratory tests	Hemoglobin^c^, g/L	129.5 (110.0, 140.5)
	Albumin^d^, g/L	38.8 (35.6, 41.8)
	Leukocytes^e^, ×10^9^/L	7.1 (5.9, 8.9)
	CRP^f^, mg/L	4.9 (2.1, 13.1)
	ESR^g^, mm/h	11.0 (7.0, 23.0)

BMI, body mass index; TNF, tumor necrosis factor; TOF, tofacitinib; CRP, C-reactive protein; ESR, erythrocyte sedimentation rate.

^a^ Disease duration was unknown in 1 patient.

^b^ BMI was unknown in 2 patients.

^c^ Hemoglobin was unknown in 8 patients.

^d^ Albumin was unknown in 13 patients.

^e^ Leukocytes was unknown in 8 patients.

^f^ CRP was unknown in 8 patients.

^g^ ESR was unknown in 17 patients.

Prior to VDZ treatment, four patients had postoperative malignant tumors, including two lung cancers, one thyroid cancer, and one breast cancer. Four patients had hepatitis B infection at baseline and were treated with antiviral drugs (two with entecavir and two with tenofovir disoproxil fumarate). Seven patients had latent tuberculosis infections, three of which were prophylactically treated with antituberculosis drugs, while the remaining four did not routinely receive antituberculosis prophylaxis. As extraintestinal manifestations, four patients had arthropathy involving the knee and hand joints, with magnetic resonance imaging showing joint effusion. Six patients had dermatological extraintestinal manifestations, including urticaria and facial acne.

### 3.2 Assessment at week 14

**FIGURE 2:**
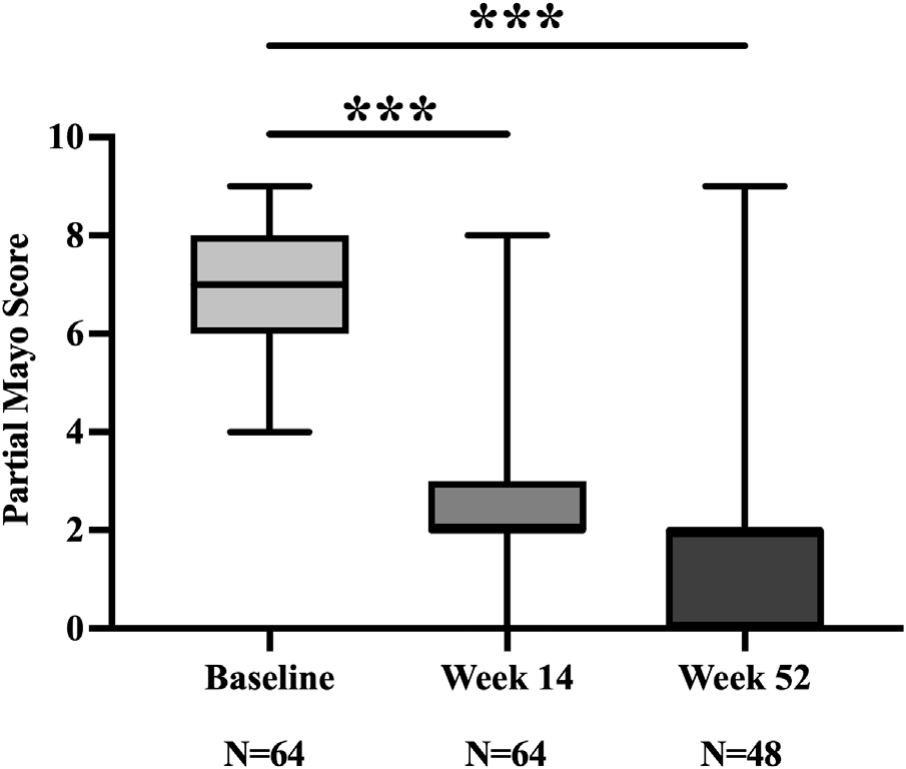
Partial Mayo Score at baseline, week 14, and week 52. ***p < 0.001.

At week 14, 73.4% (47/64) and 65.6% (42/64) of patients achieved clinical response and remission, respectively. The partial Mayo Score decreased from 7.0 (6.0, 8.0) at baseline to 2.0 (2.0, 3.0) (Figure 2). Of the 42 patients who achieved clinical remission, 7 remained on concomitant corticosteroids, yielding a steroid-free clinical remission rate of 54.7% (35/64).

**FIGURE 3:**
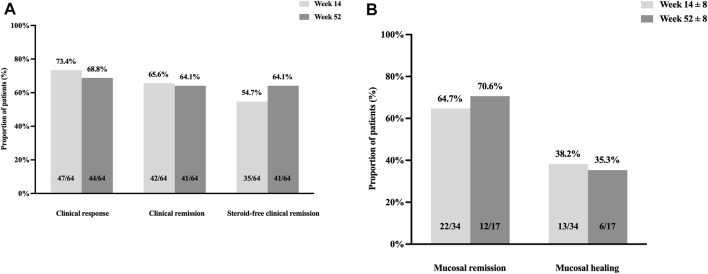
Clinical response, clinical remission, and steroid-free clinical remission at weeks 14 and 52 **(A)**. Mucosal remission and mucosal healing at weeks 14 ± 8 and 52 ± 8 **(B)**.

Thirty-four patients underwent follow-up endoscopy at week 14 ± 8, of which 64.7% (22/34) achieved mucosal remission, and 38.2% (13/34) achieved mucosal healing (Figure 3).

**FIGURE 4:**
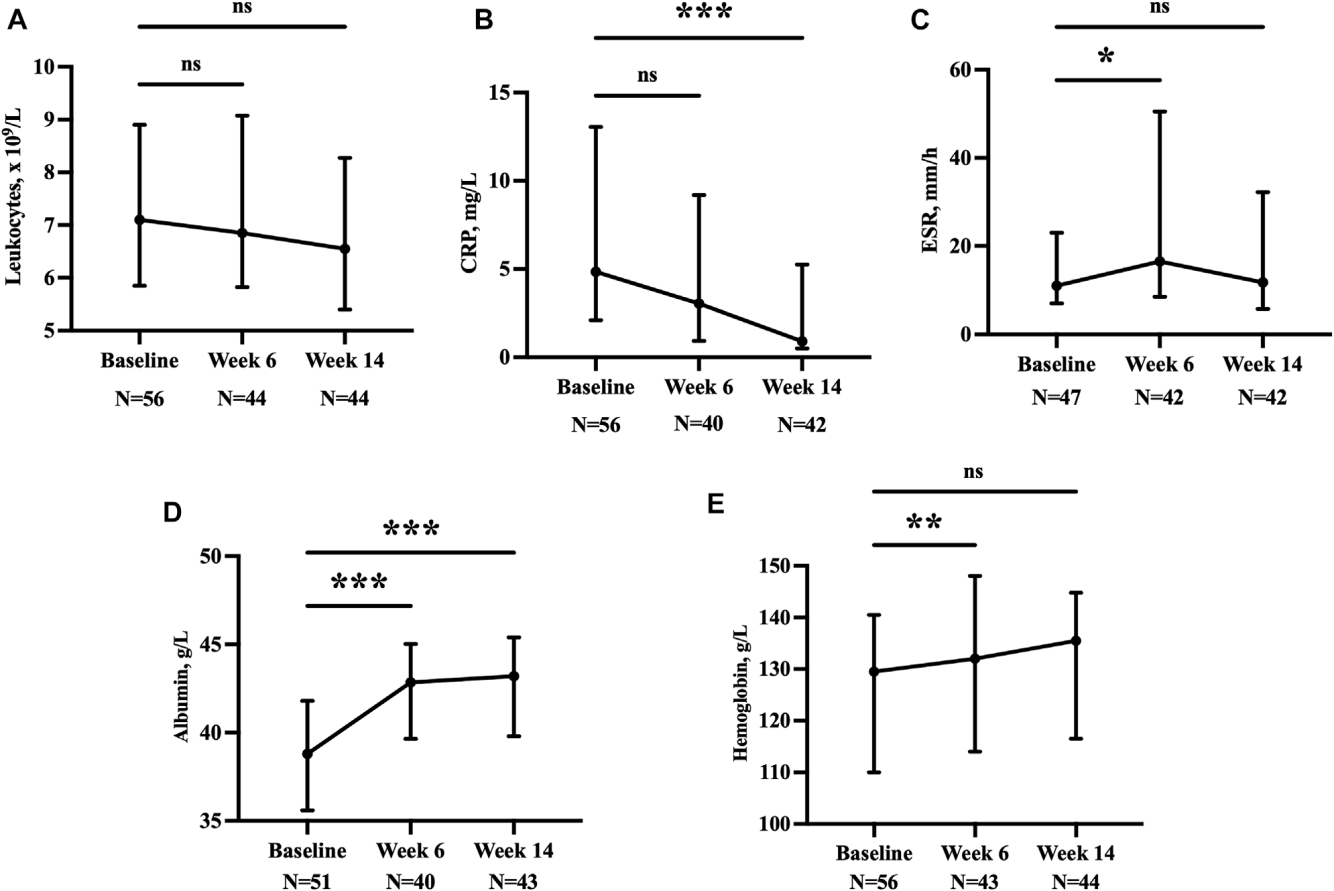
Leukocytes **(A)**, CRP **(B)**, ESR **(C)**, albumin **(D)**, and hemoglobin **(E)** at baseline, week 6, and week 14. *p < 0.05; **p < 0.01; ***p < 0.001; ns, *p* > 0.05. CRP, C-reactive protein; ESR, erythrocyte sedimentation rate.

Analysis of the laboratory test results showed that anemia and hypoproteinemia were significantly corrected at week 14 among these patients. Levels of inflammatory markers, such as leukocytes and CRP, also decreased (Figure 4).

For the 17 patients who did not achieve clinical response at week 14, their partial Mayo Score also demonstrated improvement from 7.0 (5.0, 7.5) to 5.0 (4.0, 7.0) (*p* = 0.002).

### 3.3 Assessment at week 52

Of the 47 patients showing clinical response at week 14, 46 continued VDZ treatment, with 43 having been followed up to 52 weeks. Three patients discontinued treatment due to loss of response (one discontinued after five infusions and two after seven infusions), of which one was switched to IFX, one to TOF, and one to corticosteroids. One patient did not enter the maintenance phase due to pregnancy preparation.

Of the 17 patients who did not achieve clinical response at week 14, 2 patients discontinued treatment: one was switched to IFX, and one (history of lung cancer) began chemotherapy due to an enlarged pulmonary nodule. Overall, 15 patients showed improvement in their symptoms. At Week 52, ten patients had discontinued treatment due to loss of response, including six patients after four infusions, three after six infusions, and one after seven infusions. Of the patients who discontinued treatment, two were switched to anti-TNF agents (one was administered IFX and one was administered ADA), two to TOF, two to mesalazine, two to herbal treatment, one to corticosteroids, and one to a risankizumab clinical trial.

Overall, 48 patients completed 52 weeks of treatment. Based on per protocol analysis, 91.7% (44/48) achieved clinical response, 85.4% (41/48) achieved clinical remission, and 85.4% (41/48) achieved steroid-free clinical remission. Meanwhile, intention-to-treat analysis revealed that the clinical response, clinical remission, and steroid-free clinical remission rates were 68.8% (44/64), 64.1% (41/64), and 64.1% (41/64), respectively. Seventeen patients underwent follow-up endoscopy at week 52 ± 8; 70.6% (12/17) achieved mucosal remission, and 35.3% (6/17) achieved mucosal healing (Figure 3).

### 3.4 Assessment at the end of follow-up

Among the patients who were followed up for 52 weeks, 43 continued VDZ treatment to the end of the follow-up period, of which 1 did not achieve clinical response, and concomitant use of mesalazine was initiated for symptom control. Five patients discontinued treatment, four due to loss of response and conversion to IFX, and one due to clinical remission and discontinuing VDZ on his own.

During the follow-up period, one patient underwent surgery. The patient was unresponsive to mesalazine and corticosteroids and was switched to IFX after five infusions of VDZ; the MES was 3. After two IFX infusions, the patient’s symptoms did not significantly improve, and the MES remained at 3. Finally, ileal pouch–anal anastomosis was performed.

### 3.5 Subgroup analysis

Subgroup analysis showed that the clinical response and remission rates were 78.6% and 73.2% in patients who never received anti-TNF agents, which were both higher than those in patients who had. As for treatment combined with corticosteroids, 11 patients received corticosteroids at week 14, and the clinical response and remission rates for these patients were both 72.7%; these values were not significantly different compared with those in patients treated without concomitant corticosteroids. Further analysis revealed that the clinical response and remission rates were 66.7% and 55.6% in patients aged ≥60 years, respectively, and neither value differed significantly from those in patients aged <60 years (Table 2).

**TABLE 2:** Subgroup analyses of clinical response and remission at week 14.

Stratified by		Number of patients	Clinical response	*p*-value	Clinical remission	*p*-value
Prior anti-TNF agents	Yes	8	37.5% (3/8)	**0.015**	12.5% (1/8)	**0.001**
	No	56	78.6% (44/56)		73.2% (41/56)	
Concomitant corticosteroids	Yes	11	72.7% (8/11)	0.954	72.7% (8/11)	0.589
	No	53	73.6% (39/53)		64.2% (34/53)	
Age, year	≥60	9	66.7% (6/9)	0.623	55.6% (5/9)	0.496
	<60	55	74.5% (41/55)		67.3% (37/55)	

TNF, tumor necrosis factor.

Bold values indicates that the *p* < 0.05.

### 3.6 Safety

At the end of the final follow-up visits, the proportion of patients who experienced ≥1 adverse event (AE) was 29.7% (19/64), most of which were mild. The most common AEs were skin rashes (6/64). Arthralgia, fatigue, and elevated alanine transaminase were relatively common. No cases of acute infusion reactions, delayed allergic reactions, newly onset hepatitis B or tuberculosis infection, or new malignant tumors were reported. The incidence of serious adverse events was 1.6% (1/64). The patient discontinued VDZ due to an enlarged pulmonary nodule.

At baseline, 55 patients underwent chest imaging, of whom 35 had pulmonary nodules. During the follow-up, 15 patients underwent chest imaging again. Seven patients showed no significant changes on imaging. Pulmonary nodules disappeared or decreased among four patients. Three patients had an increase in the size of the pulmonary nodule. One patient developed a new pulmonary nodule, which was diagnosed postoperatively as lung cancer. The nodule was found at week 32 after VDZ treatment, with imaging suggesting a 4-mm nodule in the upper lobe of the right lung.

## 4 Discussion

In this single-center retrospective study, analysis of data from 69 patients with UC revealed that the clinical response and remission rates after VDZ treatment were 73.4% and 65.6% at week 14% and 68.8% and 64.1% at week 52, respectively.

In Western countries, the effectiveness of VDZ for UC has been demonstrated in RCTs. For instance, the GEMINI 1 RCT showed clinical remission rates of 16.9% and 41.8% at weeks 6 and 52, respectively, significantly higher than those of the placebo (Feagan et al., 2013). By further analyzing 58 Asian participants, the findings were found to be consistent with the overall population (Ooi et al., 2021). In terms of long-term efficacy, the GEMINI LTS trial showed a clinical remission rate of 88% in patients who responded to induction therapy after a 2-year follow-up period (Loftus et al., 2017). In addition to RCTs, several RWSs from the United States, Canada, and Australia have evaluated the effectiveness of VDZ for UC. Data from one study that included nine RWSs showed that the effectiveness of VDZ for UC was similar to that reported in RCTs, with clinical remission rates of 32% and 39% at weeks 14 and 52, respectively (Engel et al., 2018). For Asian populations, few RWSs have been conducted, with most performed in Korea and Taiwan (Table 3). In the VIOLET study conducted in Taiwan, the clinical response, clinical remission, and mucosal healing rates at the 1-year follow-up were 76.0%, 58.0%, and 62.2%, respectively (Lin et al., 2023). In comparison, the clinical response and remission rates were higher in our study, which may be attributed to the larger number of patients who had not previously received anti-TNF agents (84.1%).

**TABLE 3:** Outcomes of real-world studies on vedolizumab for UC in Asia.

Reference	Time	Region	Type	Characteristic	Outcome
Gan et al. (2019)	2019	Singapore	Abstract	25 UC 17 had received anti-TNF agents	Steroid-free clinical remission at weeks 14, 24, and 54: 68.0%, 66.7%, and 80.0% Mucosal remission at week 31: 35.3%
Tai et al. (2019)	2019	Taiwan	Abstract	8 UC	Clinical response and clinical remission at month 6: 87.5% (7/8) and 25.0% (2/8)
Chiu et al. (2021)	2019	Taiwan	Article	9 UC	Clinical response, clinical remission, and steroid-free clinical remission at week 14: 100% (7/7), 0 (0/7) and 40% (2/5) Mucosal response and mucosal remission at week 14: 85.7% (6/7) and 0 (0/7)
Kim et al. (2021)	2021	Korea	Article	78 UC Failure of prior anti-TNF agents	Clinical response, clinical remission, steroid-free clinical remission at week 14: 68.0%, 44.0% and 40.0% Mucosal response and mucosal remission at week 14: 54.4% and 32.4%
Oh et al. (2021)	2021	Korea	Abstract	84 UC Failure of prior anti-TNF agents	Clinical response, clinical remission, and steroid-free clinical remission at week 54: 45.7%, 41.4%, and 37.1% Mucosal remission at week 54: 27.1%
Ye et al. (2021)	2021	Korea	Article	105 UC Failure of prior anti-TNF agents	Clinical response and clinical remission at week 14: 73.2% and 39.4%
Kuo et al. (2022)	2021	Taiwan	Article	37 UC 7 had received anti-TNF agents	Clinical response and clinical remission at weeks 8–10: 56.8% and 32.4% Mucosal remission at weeks 8–10: 58.3%
Lin et al. (2023)	2023	Taiwan	Article	147 UC 70.7% were biologic-naïve	Clinical response, clinical remission, steroid-free clinical remission, and mucosal healing at 1 year: 76.0%, 58.0%, 35.0%, and 62.2%

UC, ulcerative colitis; TNF, tumor necrosis factor.

Notably, 84.1% of patients in our study had not previously received anti-TNF agents, which is higher than studies such as GEMINI 1 (59.0%) and VIOLET (70.7%). However, we included consecutive UC patients who used VDZ at our center strictly according to the inclusion criteria and did not exclude patients who had previously received anti-TNF agents. A possible reason for this discrepancy at baseline is the consensus of Chinese experts that VDZ may be used as first-line treatment against moderate to severe UC, especially for those with early onset, severe disease, rapid progression, and poor prognosis. In addition, hepatitis B and tuberculosis infections are prevalent in many Asian countries, including China (Banerjee et al., 2020); VDZ has a favorable safety profile and has not been reported to increase the risk of reactivation of these diseases (Ng et al., 2018). Finally, the inclusion of VDZ in the Chinese national basic medical insurance in March 2021 might have led to more patients choosing this drug as first-line therapy.

In this study, VDZ showed satisfactory safety profiles. During the follow-up period, no patient reported acute infusion reactions, delayed allergic reactions, or infections. Progressive multifocal leukoencephalopathy was not reported, consistent with the results of the GEMINI LTS trial (Loftus et al., 2020). Analysis of the 4-year post-marketing data using the VDZ Global Safety Database revealed that the most frequently reported adverse events were gastrointestinal events, and <1% of the patients reported malignancies (Cohen et al., 2020b). Overall, the frequency of adverse events was low, and most were non-serious. However, after treatment with VDZ, some patients in our study developed skin and joint manifestations, which must be considered by physicians. An RWS based on the OBSERV-IBD cohort also found that inflammatory arthropathy was observed in 34 (13.8%) of the 247 patients treated with VDZ (Tadbiri et al., 2018). Another study that included 112 patients also observed joint manifestations in 17 patients (15.2%) (Dupré et al., 2020).

Patients with different baseline characteristics may have different outcomes. In a meta-analysis that included 79 clinical trials, patients who did not receive biologics were more likely to achieve clinical remission at week 52 than those who had previously received biologics (relative risk [RR] = 1.32, 95% CI 1.14–1.53). Additionally, patients who did not receive biologics had a lower risk for serious adverse events (RR = 0.29, 95% CI 0.09–0.95) (Attauabi et al., 2022). Our study found that patients who had not previously received anti-TNF agents were more likely to achieve clinical response and remission at week 14 than those who had, demonstrating the advantages of VDZ as first-line biological therapy. However, the result should be interpreted with caution. First, subgroup analyses are *post hoc* analyses that cannot maintain randomization within the subgroup; second, the small sample size of patients who had not previously received anti-TNF agents may lead to false positives. Therefore, the results must be validated in subsequent clinical trials.

There is also a growing interest in the effectiveness and safety of VDZ in the elderly. Cohen et al. conducted a multicenter retrospective cohort study and found that patients with UC aged <40 and >60 years had similar clinical and endoscopic responses after a year of VDZ treatment (Cohen et al., 2020a). In another case-control study, similar findings were obtained, with no significant differences in mucosal healing between patients with UC aged ≥65 and <65 years (Shashi et al., 2020). The 2021 update of the AGA clinical guidelines states that VDZ has similar efficacy in older and younger patients, and the incidence of adverse events is not significantly correlated with age (Ananthakrishnan et al., 2021). Besides, VDZ tends to be used more frequently than other biological agents in older patients who are more likely to develop complications. Although there were only 11 elderly patients in the subgroup analysis in our study, nearly two-thirds achieved clinical remission at week 14, suggesting that VDZ is effective in elderly patients.

Currently, the combination of VDZ with small-molecule drugs is an option for patients who are unresponsive to first-line biological therapy. Goessens et al. reported 12 patients who received a combination of VDZ and TOF, eight of whom (67%) achieved endoscopic response after 11 months (Goessens et al., 2021). A case report by Bonastre et al. also showed improvement in the levels of fecal calprotectin, CRP, and other parameters with the combination of VDZ and TOF (Taberner Bonastre et al., 2021). In our study, two patients were treated with a combination of VDZ and TOF, but the results were unsatisfactory. The patients discontinued VDZ after four and seven infusions, respectively, due to loss of response. A recent meta-analysis suggested that dual biological or small-molecule therapy may be effective in patients with IBD. However, the study integrated various combinations of biologics and did not analyze the results of combinatorial VDZ and TOF alone (Ahmed et al., 2022). In fact, no large clinical trial has explored outcomes and prognosis while considering the combination of these two drugs.

Our study has certain limitations. First, data were obtained retrospectively and may have been subject to bias. Second, the small sample size did not allow for the analysis of risk factors affecting effectiveness, and we may have missed recording adverse events with a low probability of occurrence. Third, not all patients underwent follow-up endoscopy at the end of the induction phase and after 1 year, and not all patients who underwent follow-up endoscopy were concentrated on these two periods. Therefore, the time frame for the endoscopic endpoint follow-up was expanded in our study.

In summary, our findings indicate that the effectiveness of long-term use of vedolizumab for Chinese patients with UC was generally similar to that previously reported in other regions and populations. Patients who had not previously received anti-TNF agents may have better outcomes than those who had in the induction phase. More studies are warranted to explore the effectiveness and safety of VDZ in patients with different baseline characteristics and to investigate the combination of VDZ with other biological agents, which will help physicians to make better treatment decisions for patients with complex IBD.

## Data Availability

The raw data supporting the conclusion of this article will be made available by the authors, without undue reservation.
